# Cross-border data sharing for research in Africa: an analysis of the data protection and research ethics requirements in 12 jurisdictions

**DOI:** 10.1093/jlb/lsaf002

**Published:** 2025-03-19

**Authors:** Ciara Staunton, Aliki Edgcumbe, Lukman Abdulrauf, Amy Gooden, Paul Ogendi, Donrich Thaldar

**Affiliations:** Institute for Biomedicine, Eurac Research, Bolzano, Italy; School of Law, University of KwaZulu-Natal, Durban, South Africa; School of Law, University of KwaZulu-Natal, Durban, South Africa; School of Law, University of KwaZulu-Natal, Durban, South Africa; Department of Public Law, University of Ilorin, Ilorin, Nigeria; School of Law, University of KwaZulu-Natal, Durban, South Africa; School of Law, University of KwaZulu-Natal, Durban, South Africa; Faculty of Law, University of Nairobi, Kenya; School of Law, University of KwaZulu-Natal, Durban, South Africa

**Keywords:** privacy, data protection, data sharing, data governance, ethics, adequacy, consent

## Abstract

**Background:**

In recent years, there has been a notable uptake in genomic and health-related research activities across the African continent. Similarly, there has been increased introduction of data protection legislation that affects the sharing of personal data, such as health data and genomic data, including for research. Many of these statutes have stricter requirements when sharing personal data across borders. Consequently, the cross-border sharing of health data, that includes genetic data, requires careful navigation of the pertinent data protection legislation, in particular concerning the sharing of such data for research purposes. To help researchers navigate these legal frameworks, 12 African countries were analysed to develop country guides on cross-border data sharing.

**Results:**

Of the 12 African countries that were analysed, 10 have data protection laws in place (Botswana, Ghana, Kenya, Malawi, Nigeria, Rwanda, South Africa, Tanzania, Uganda, and Zimbabwe), while two countries (Cameroon and The Gambia) do not. (At the time of the study, Cameroon did not have a data protection regulation in place. Law No. 2024/017 on the Protection of Personal Data is now in force.) With the exception of Ghana, all countries with data protection statutes had additional requirements to be met when sharing personal data across borders. Consent and adequacy are the most common grounds for justifying the sharing of personal data across borders.

**Conclusion:**

Given the limitations of the current models of consent, consent is not a suitable basis to transfer large quantities of data for research. Adequacy is a common ground, but there are national differences in the implementation of this ground. Researchers must therefore analyse each national legal framework and make decisions on a case-by-case and country-by-country basis.

## I. BACKGROUND

In recent years, there has been a notable uptake in genomic and health-related research activities across Africa, driven in part by the collaborative efforts of national and international consortia such as Human Heredity and Health in Africa (H3Africa), Bridging Biobanking and Biomedical Research Across Europe and Africa (B3Africa), the Public Health Alliance for Genomic Epidemiology (PHA4GE), and the recently established Data Science for Health Discovery and Innovation in Africa (DS-I Africa). This research entails collecting, using, and disseminating genomic and other health-related data, with a significant emphasis on cross-border data sharing. While the collaborative sharing of such data is indispensable for advancing scientific knowledge, it also highlights a host of legal and ethical considerations that demand careful attention.^1^

Some of these ethico-legal issues relate to the ongoing debates about appropriate consent models for this research. While broad consent enables data to be shared for secondary research purposes more easily, it does not account for the individual preferences of research participants, which may evolve over time,^2^ and there are also concerns that broad consent may not be permitted under some national data protection legislation.^3^ Static models of consent do not easily provide for these changing preferences. Accordingly, other models of consent, such as dynamic consent, which uses information technology to enable participants to change and update their preferences, have been proposed as alternatives.^4^ There are increasing numbers of examples of the successful use and implementation of dynamic consent in high-income countries (HICs) but, to date, there are a paucity of examples in the African context.^5^

Beyond consent-related issues, there are concerns that the data may be used to stigmatize or discriminate against individuals or their communities.^6^ The use and sharing of health data that includes genetic data must have processes in place to guard against and mitigate such risks. Concerns about the exportation of samples and data for research stem from past exploitative practices, often referred to as ‘parachute research’. This term describes instances where samples and data were collected in low- and middle-income countries (LMICs) without local oversight and the data were subsequently sent to HICs for research purposes.^7^

Most recently, however, the greatest attention has been given to the risk to privacy in the use of health data that includes genetic data.^8^ This heightened awareness and scrutiny is partly due to the introduction of national data protection legislation across the globe that seeks to regulate the processing—including collection, storage, use, and sharing—of personal data, and health data that includes genetic data. Research teams and consortia accessing, using, and sharing health data must now navigate the data protection regulations and consider the impact that these regulations will have on their research.

Similar legislative development in the context of data protection has occurred in Africa. Regionally, there was the introduction by the African Union (AU) of the Malabo Convention (formally known as the *African Union Convention on Cyber Security and Personal Data Protection*) in 2014 in Malabo, Equatorial Guinea.^9^ The convention’s primary purpose is to establish a legal framework to promote cybersecurity, cybercrime prevention, and the protection of personal data within the African continent. To date, over 30 African countries have introduced data protection legislation. Therefore, the growth of genomic and health-related research comes at a time when data protection legislation is strengthening across the continent. Such legislation comprises general legal frameworks in that they apply to the processing of all personal data, including health data that includes genetic data, in the research context. The legislation sets out the conditions that must be met in the processing of personal data, the rights of data subjects, the responsibilities of the various individuals involved in the processing of personal data, and the security requirements that must be met, among others. Most data protection legislation does, however, have special provisions in place for research. These may include exemptions to some of the strict processing requirements and exemptions to some of the rights provided for data subjects if the processing is for research. In contrast, many legislative frameworks have stricter requirements when processing genetic data. Such legislation generally also has specific requirements that must be met when transferring personal data across borders. Consequently, the cross-border sharing of health data, that includes genetic data, requires the careful analysis of the pertinent data protection legislation, in particular concerning the sharing of such data for research purposes.

The DS-I Africa Initiative was established in 2021 in order to leverage data science technologies to transform biomedical and behavioural research and to develop solutions to improve individual and population health. A key activity of the research involves the sharing of health data that includes genetic data. The DS-I Africa Law project focuses on the legal dimensions of using data science for health discovery and innovation in Africa and aimed to provide scientists with the necessary guidance on how to be legally compliant. The project has a broad jurisdictional scope, involving the law of 12 African nations: Botswana, Cameroon, Ghana, Kenya, Malawi, Nigeria, Rwanda, South Africa, Tanzania, The Gambia, Uganda, and Zimbabwe. These countries were selected based on the criteria of having hosted H3Africa projects in the past, and having available legislation in English. Five critical legal themes were investigated: (i) modes of informed consent to the use of data; (ii) the nature and content of individual and community rights in genomic data; (iii) the use of persons’ geospatial data for public health surveillance; (iv) the cross-border sharing of data; and (v) the use of data as a basis for artificial intelligence (AI). To assist researchers in understanding the impact of national data protection legislation on the cross-border sharing of data, the DS-I Africa Law project sought to develop country guides for 12 jurisdictions on the cross-border sharing of data. This paper reports on the findings of those guides.

The aim of this paper is two-fold: First to present the requirements of each country in the cross-border sharing of data, and second, to offer a comparative analysis of the requirements. This paper will demonstrate that, while on the face of it, there are many similarities in the requirements to be met when sharing data across borders in the 12 countries, the differences are such that there is a lack of a harmonized approach in how health data can be shared across borders for research. This does mean that researchers must navigate the legal requirements of each jurisdiction to identify a suitable legal basis on which it may transfer health data.

This paper begins by outlining the methodology adopted to develop the above country guides. It then discusses how these country guides were analysed. This paper then discusses the key findings, including a comparison of the key features of the applicable data protection legislation, the differing grounds across the 12 jurisdictions through which personal data may be transferred, and the requirements under the national research ethics and research regulatory documents that must be met when transferring personal data across borders for research. Finally, this paper offers some reflections on the implications of these findings in these 12 countries when sharing health data that includes genetic data across borders for research purposes.

## II. METHODS

Two authors of this paper (CS & DT) developed a template guide (Appendix A) drawing from the General Data Protection Regulation 2016/679 (GDPR) in the European Union (EU). The GDPR was used as most data protection legislation in Africa are based on the GPDR. In developing the template guide, it was decided to focus on both research ethics regulatory frameworks and data protection to ensure that researchers have a comprehensive overview of the national requirements to be met when sharing health data that includes genetic data across borders.

For the national research ethics frameworks, the template required all applicable national laws, regulations, and guidelines to be stated, followed by detail of all national requirements that must be met in cross-border data sharing. The template then focused on data protection-related issues. Although the purpose of the guides was to provide detailed guidance on the additional requirements for the cross-border sharing of data, the authors considered it important to contextualize this information and provide researchers with basic information on the data protection legislation generally. The template first required details on whether the relevant country had signed or ratified the Malabo Convention that came into force in June 2023. Next, the template set out the details to be filled in as they relate to the application of the national data protection law, the definition of different categories of data, a definition and description of the key individuals, the principles to be met in the processing of personal data, the rights of the data subjects and, finally, the grounds under which personal data can be shared across borders.

Once the template was finalized, training on the use of the template was provided to the research assistants (RAs). The RAs then inputted the relevant data for the 12 countries. CS checked this work and sent back queries and points for clarification. This continued until all issues were addressed. The draft country guides were then sent to the country experts (LA, AA, AG, and PO) for review. Following this, CS reviewed these edits and sent back queries and points for clarification. This continued until all issues had been addressed. Finally, AE reviewed the sections on the application of data protection legislation and made changes where necessary.

On completion of the country guides, CS compared each country guide under the following criteria: Application of data protection law, descriptors of categories of data, descriptors of relevant individuals specified in the data protection law, requirements for the processing of personal data, rights of data subjects, grounds for the cross-border flow of data, and additional requirements from research ethics regulatory frameworks.

Following this, the country guides were compared to identify similarities and differences between each jurisdiction. This information is correct as of September 2024.

### II.A. Comparative Analysis

Out of the 12 countries, two countries (Cameroon^10^ and The Gambia) have no data protection legislation in place. The remaining 10 countries have a data protection statute in force. As represented in Table 1, two countries (Ghana and Rwanda) have ratified the Malabo Convention, while three other countries (Cameroon, South Africa, and The Gambia) have signed the Malabo Convention.

**Table 1:** Ratification and signing of Malabo Convention

**Country**	**Ratified**	**Signed**
Botswana	No	No
Cameroon	No	Yes
Ghana	Yes	Yes
Kenya	No	No
Malawi	No	No
Nigeria	No	No
Rwanda	Yes	Yes
South Africa	No	Yes
Tanzania	No	No
The Gambia	No	Yes
Uganda	No	No
Zimbabwe	No	No

#### 1. Application of data protection law

In all 10 of the above countries, the data protection legislation applies only to personal data. Notably, Zimbabwe incorporates provisions that extend to non-personal data. While some countries provide additional guidance, the usual approach to determining whether data falls under data protection legislation is whether it meets the definition of personal data.

In South Africa, information is considered personal when it relates to an identified or identifiable person. It is this personal information that data protection law seeks to protect. De-identified data is explicitly excluded from the Protection of Personal Information Act 4 of 2013 (POPIA) and does not concern or apply to de-identified non-personal information. De-identifying information is the process of stripping the data of any information which can be used to identify a data subject. It should not be possible to re-identify the data subject directly or indirectly by manipulating the information or linking it with other information.

Some legislation does mention anonymized and pseudonymized data. In Kenya, although neither Kenya’s Data Protection Act nor its Regulations explicitly exclude anonymized data from the ambit of its provisions, according to the Act, ‘anonymization’ means ‘the removal of personal identifiers from personal data so that the data subject is no longer identifiable’. The Act provides that data must be anonymized to ensure ‘the data subject is no longer identifiable’. Unfortunately, Kenya’s Data Protection Act and the Kenyan Data Protection General Regulations do not contain standards for non-identifiability.

In Rwanda the Law Relating to the Protection of Personal Data and Privacy 2021 does refer to ‘de-identified’ data and ‘pseudonymization’. Pseudonymization is when information is removed from the data so it is not possible to identify an individual, and that information is kept separate through technical and organizational measures. Similar to the GDPR, it is clear that data that has been pseudonymized does fall under the Act. ‘De-identified’ data is mentioned in the Act, but is not defined. Article 57 provides that it is an offence to knowingly, recklessly, or intentionally re-identify data that has been de-identified. It appears from this context that de-identification is a reversible technique.

Tanzania’s Data Protection Act does not refer to anonymized or pseudonymized data, but the Personal Data Protection (Personal Data Collection and Processing) Regulations 2023, refer to both anonymization and pseudonymization, but they are not defined. From the context in which it is used, anonymization is a tool that may be employed by data controllers or data processors to minimize their use or retention of data in an identifiable form where it is not necessary to do so. This aligns with the principles of proportionality, necessity, retention, and storage of personal data. The data controller or data processor must ensure that there is ‘*no possibility* of re-identification of anonymous personal data’ and that this is properly tested (emphasis added, regulation 30(d)). The inclusion of the phrase ‘no possibility’ and the requirement for this to be tested suggests that for data to be considered anonymized, the anonymization must be proved through testing to be effective and absolute. Although not defined, pseudonymization is referred to as a safety measure that involves ‘storing identification keys separately’ (regulation 28(d)).

In Nigeria, the Data Protection Act (NDPA) applies to personal data but does refer to de-identification and pseudonymization. Data that has been pseudonymized does fall under the NDPA. Although ‘de-identification’ is mentioned in the NDPA, it is not defined. From its context in section 39, de-identification is one of the technical and organizational measures a data controller may use to ensure the security, integrity and confidentiality of the personal data under its control, in order to guard against misuse, or unauthorized disclosure or access, among others.

The Data Protection Act 2024 in Malawi refers to ‘de-identification’ and ‘pseudonymization’. Data that has been pseudonymized remains in the ambit of the Data Protection Act. Although ‘de-identification’ is mentioned in the Act, it is not defined. From its context in section 31(2), de-identification is one of the technical and organizational measures a data controller may use to ensure the security, integrity, and confidentiality of the personal data in its control, in order to guard against misuse, or unauthorized disclosure or access, among others. In the absence of specific guidance and clarity in the alternative on this point, it would seem that data that has been de-identified falls squarely within the ambit of the Act.

#### 2. Defining personal data and sensitive data

Table 2 lists the definitions for personal data and sensitive personal data. Personal data is typically data about a particular person that can identify them. In South Africa, this is referred to as ‘personal information’. Sensitive personal data pertains to information that is particularly sensitive in respect of an individual, such as health or genetic data. This category of data receives special protection under data protection legislation and bills. In South Africa, it is called ‘special personal information’.

**Table 2:** Personal data and sensitive data

**Country**	**Personal data**	**Sensitive personal data**
*Botswana*	Information relating to an identified or identifiable individual, which individual can be identified directly or indirectly, in particular by reference to an identification number, or to one or more factors specific to the individual’s physical, physiological, mental, economic, cultural or social identity; and ‘data’ shall be construed accordingly. (Data Protection Act, 2018 s2)	Personal data relating to a data subject which reveals his or her – (a) racial or ethnic origin; (b) political opinions; (c) religious beliefs or philosophical beliefs; (d) membership of a trade union; (e) physical or mental health or condition; (f) sexual life; (g) filiation; or (h) personal financial information, and includes – (a) any commission or alleged commission of him or her of any offence; (b) any proceedings for any offence committed or alleged to have been committed by him or her, the disposal of such proceedings, or the sentence of any court in such proceedings; and (c) genetic data, biometric data and the personal data of minors. (Data Protection Act, 2018 s2)
*Kenya*	Means any information relating to an identified or identifiable natural person. (The Data Protection Act, 2019 s2)	Means data revealing the natural person’s race, health status, ethnic social origin, conscience, belief, genetic data, biometric data, property details, marital status, family details including names of the person’s children, parents, spouse or spouses, sex or the sexual orientation of the data subject. (The Data Protection Act, 2019 s2)
*Malawi*	Any data relating to an identifiable natural person which, directly or indirectly, by reference to an identifier such as a name, an identification number, location data, an online identifier or one or more factors specific to the physical, physiological, genetic, psychological, cultural, social or economic identity of that person. (Data Protection Act, 2024 s2)	Personal data relating to a natural person’s – (a) biometric data; (b) race or ethnic origin; (c) religious or other belief relating to the freedom of conscience of the person; (d) health status; (e) political opinion or affiliation; and (f) such other data as the Minister may prescribe. (Data Protection Act, 2024 s2)
*Nigeria*	Any information relating to an individual, who can be identified or is identifiable, directly or indirectly, by reference to an identifier such as a name, an identification number, location data, an online identifier or one or more factors specific to the physical, physiological, genetic, psychological, cultural, social, or economic identity of that individual (Nigeria Data Protection Act, 2023 s65)	Personal data relating to an individual’s – (a) genetic and biometric data, for the purpose of uniquely identifying a natural person; b) race or ethnic origin; (c) religious or similar beliefs, such as those reflecting conscience or philosophy; (d) health status; (e) sex life; (f) political opinions or affiliations; (g) trade union memberships; or h) other information prescribed by the Commission, as sensitive personal data under section 30 (2); and ‘social security laws’ means the Employee Compensation Act, Pension Reform Act, National Health Insurance Authority Act, National Housing Fund Act, Nigeria Social Insurance Trust Fund Act, Industrial Trust Fund Act or any other similar law. (Nigeria Data Protection Act, 2023 s65)
*Rwanda*	Information relating to an identified or identifiable natural person who can be identified, directly or indirectly, in particular by reference to an identifier such as a name, an identification number, location data, an online identifier or to one or more factors specific to the physical, psychological, genetic, mental, economic, cultural or social identity of that natural person. (Law relating to the protection of personal data and privacy Article 3)	Information revealing a person’s race, health status, criminal records, medical records, social origin, religious or philosophical beliefs, political opinion, genetic or biometric information, sexual life or family details. (Law relating to the protection of personal data and privacy Article 3)
*South Africa*	Personal information means information relating to an identifiable, living, natural person, and where it is applicable, an identifiable, existing juristic person, including, but not limited to – (a) information relating to the race, gender, sex, pregnancy, marital status, national, ethnic or social origin, colour, sexual orientation, age, physical or mental health, well-being, disability, religion, conscience, belief, culture, language and birth of the person; (b) information relating to the education or the medical, financial, criminal or employment history of the person; (c) any identifying number, symbol, e-mail address, physical address, telephone number, location information, online identifier or other particular assignment to the person; (d) the biometric information of the person; (e) the personal opinions, views or preferences of the person; (f) correspondence sent by the person that is implicitly or explicitly of a private or confidential nature or further correspondence that would reveal the contents of the original correspondence; (g) the views or opinions of another individual about the person; and (h) the name of the person if it appears with other personal information relating to the person or if the disclosure of the name itself would reveal information about the person (Protection of Personal Information Act No. 4 of 2013 s1)	Special personal information means personal information as referred to in section 26: ‘A responsible party may, subject to section 27, not process personal information concerning – the religious or philosophical beliefs, race or ethnic origin, trade union membership, political persuasion, health or sex life or biometric information of a data subject […]’ (Protection of Personal Information Act No. 4 of 2013 s26)
*Tanzania*	Means data about an identifiable person that is recorded in any form, including – (a) personal data relating to the race, national or ethnic origin, religion, age or marital status of the individual; (b) personal data relating to the education, the medical, criminal or employment history; (c) any identifying number, symbol or other particular assigned to the individual; (d) the address, fingerprints or blood type of the individual; (e) the name of the individual appearing on personal data of another person relating to the individual or where the disclosure of the name itself would reveal personal data about the individual; (f) correspondence sent to a data controller by the data subject that is explicitly or implicitly of a private or confidential nature, and replies to such correspondence that would reveal the contents of the original correspondence, and the views or opinions of any other person about the data subject (Personal Data Protection Act 2022 s3)	Sensitive personal data includes – (a) genetic data, data related to children, data related to offences, financial transactions of the individual, security measures or biometric data; (b) if they are processed for what they reveal, personal data revealing racial or ethnic origin, political opinions, religious or philosophical beliefs, affiliation, trade-union membership, gender and data concerning health or sex life; and (c) any personal data otherwise considered under the laws of the country as presenting a major risk to the rights and interests of the data subject (Personal Data Protection Act 2022 s3)
*Uganda*	Information about a person from which the person can be identified, that is recorded in any form and includes data that relates to – (a) the nationality, age or marital status of the person; (b) the education level or, occupation of the person; (c) an identification number, symbol or other particulars assigned to a person; (d) identity data; or (e) other information which is in the possession of, or is likely to come into the possession of the data controller and includes an expression of opinion about the individual. (Data Protection and Privacy Act, 2019 s2)	Not defined. However, s9 provides that special personal data relates to religious or philosophical beliefs, political opinion, sexual life, financial information, health status or medical records of an individual (Data Protection and Privacy Act, 2019 s9)
*Zimbabwe*	Information relating to a data subject, and includes – (a) the person’s name, address or telephone number; (b) the person’s race, national or ethnic origin, colour, religious or political beliefs or associations; (c) the person’s age, sex, sexual orientation, marital status or family status; (d) an identifying number, symbol or other particulars assigned to that person; (e) fingerprints, blood type or inheritable characteristics; (f) information about a person’s health care history, including a physical or mental disability; (g) information about educational, financial, criminal or employment history; (h) opinions expressed about an identifiable person; (i) the individual’s personal views or opinions, except if they are about someone else; and (j) personal correspondence pertaining to home and family life. (Data Protection Act 2021 [*Chapter 11:22*] s3)	Information or any opinion about an individual which reveals or contains the following – (a) racial or ethnic origin; (b) political opinions; (c) membership of a political association; (d) religious beliefs or affiliations; (e) philosophical beliefs; (f) membership of a professional or trade association; (g) membership of a trade union; (h) sex life; (i) criminal, educational, financial or employment history; (j) gender, age, marital status or family status; health information about an individual; genetic information about an individual; or any information which may be considered as presenting a major risk to the rights of the data subject. (Data Protection Act 2021 [*Chapter 11:22*] s3)

In Zimbabwe, there is an additional category of data covered in its Data Protection Act, which is referred to as ‘data’. This is defined as ‘any representation of facts, concepts, information, whether in text, audio, video, images, machine-readable code or instructions, in a form suitable for communications, interpretation or processing in a computer device, computer system, database, electronic communications network or related devices and includes a computer programme and traffic data’. This category of data does not appear in any other data protection act.

#### 3. Key role-players in data protection legislation

All the acts allocate rights and duties to roughly the same set of key role-players. These role-players are typically referred to as a ‘data subject’, a ‘data processor’, a ‘data controller’, and a ‘data protection officer’. Table 3 provides the exact definition in each country. Typically, a data subject is the person to whom the personal data relates. A data controller is generally the person who decides what the data will be used for. In the research context, this will be the person deciding on the purpose of the research and how it will be achieved. Legal responsibility generally falls on the Principal Investigator (PI) and the institution as the employer.^11^

**Table 3:** Key role-players in the data protection legislation

**Country**	**Data subject**	**Data processor**	**Data controller**	**Data Protection Officer**
*Botswana*	An individual who is the subject of personal data. (Data Protection Act, 2018 s2)	A person who processes data on behalf of the data controller. (Data Protection Act, 2018 s2)	A person who alone or jointly with others determines the purposes and means of which personal data is to be processed, regardless of whether or not such data is processed by such person or agent on that person’s behalf. (Data Protection Act, 2018 s2)	A person who is appointed by the data controller, which person shall independently ensure that personal data is processed in a correct and lawful manner (called a Data Protection Representative). (Data Protection Act, 2018 s2)
*Kenya*	Means an identified or identifiable natural person who is the subject of personal data. (The Data Protection Act, 2019 s2)	Means a natural or legal person, public authority, agency or other body which processes personal data on behalf of the data controller. (The Data Protection Act, 2019 s2) The Data Protection Act, 2019 s42 (3) Where a data processor processes personal data other than as instructed by the data controller, the data processor shall be deemed to be a data controller in respect of that processing.	Means a natural or legal person, public authority, agency or other body which, alone or jointly with others, determines the purpose and means of processing of personal data. (The Data Protection Act, 2019 s2)	Not defined in the Act. However, it is appointed in terms of section 24 of the Act. S24 (7): A data protection officer shall – (a) advise the data controller or data processor and their employees on data processing requirements provided under this Act or any other written law; (b) ensure on behalf of the data controller or data processor that this Act is complied with; (c) facilitate capacity-building of staff involved in data processing operations; (d) provide advice on data protection impact assessment; and (e) cooperate with the Data Commissioner and any other authority on matters relating to data protection. Chatbot: A data protection officer, as per the context, is an individual who can be designated or appointed by a data controller or data processor under certain terms and conditions. This designation or appointment can occur when the processing is carried out by a public or private body (excluding courts acting in their judicial capacity), or when the core activities of the data controller or processor involve regular and systematic monitoring of data subjects or processing of sensitive categories of personal data. The Data Protection Officer can be a staff member of the data controller or processor and can fulfil other tasks and duties provided they do not result in a conflict of interest. A group of entities may appoint a single Data Protection Officer, provided that such officer is accessible by each entity. A person may be designated as a Data Protection Officer if they have relevant academic or professional qualifications, including knowledge and technical skills in matters relating to data protection. The data protection Officer’s duties include advising the data controller or processor and their employees on data processing requirements, ensuring compliance with the Act, facilitating capacity-building of staff involved in data processing operations, providing advice on data protection impact assessment, and cooperating with the Data Commissioner and other authorities on matters relating to data protection. (The Data Protection Act, 2019 s24)
*Malawi*	A natural person to whom particular personal data relates. (Data Protection Act, 2024 s2)	A natural or legal person who processes personal data on behalf of a data controller. (Data Protection Act, 2024 s2)	A natural or legal person who, alone or jointly with another natural or legal person, determines the purpose and means of processing personal data. (Data Protection Act, 2024 s2)	A person designated as such pursuant to section 33. (Data Protection Act, 2024 s2)
*Nigeria*	An individual to whom personal data relates (Nigeria Data Protection Act, 2023 s65)	An individual, private entity, public authority or any other body, who/which processes personal data on behalf of or at the direction of a data subject (Nigeria Data Protection Act, 2023 s65)	An individual, private entity, public Commission, agency or any other body who/which, alone or jointly with others, determines the purposes and means of processing of personal data (Nigeria Data Protection Act, 2023 s65)	A designated individual with expert knowledge of data protection law and practices. This person has the ability to carry out tasks prescribed under the Act and subsidiary legislation made under it. He/she may be an employee of a data controller or engaged by a service contract. Their responsibilities include advising the data controller or the data processor and their employees, monitoring compliance with the Act and related policies of the data controller or data processor, and acting as the contact point for the Commission on issues relating to data processing. (Nigeria Data Protection Act, 2023 s32)
*Rwanda*	A natural person from whom or in respect of whom personal data has been requested and processed. (Law relating to the protection of personal data and privacy Article 3)	Natural person, public or private corporate body or legal entity, who/which is authorized to process personal data on behalf of the data controller. (Law relating to the protection of personal data and privacy Article 3)	A natural person, public or private corporate body or legal entity who/which, alone or jointly with others, processes personal data and determines the means of their processing. (Law relating to the protection of personal data and privacy Article 3)	A person designated by the data controller or the data processor or associations and other bodies representing categories of data controllers or data processors in accordance with the provisions of the law. This officer is designated on the basis of professional qualities, expert knowledge of personal data protection, practices, and the ability to fulfil the tasks assigned to him or her. The Personal Data Protection Officer may be a permanent staff member of the data controller or the data processor, or a person who fulfils the tasks on the basis of a service contract. The officer’s role includes due regard to the risk associated with personal data processing operations, considering the nature, scope, context and purpose of processing. (Law relating to the protection of personal data and privacy Article 40)
*South Africa*	The person to whom personal information relates (Protection of Personal Information Act, 2013 s1)	*Called an operator*, meaning a person who processes personal information for a responsible party in terms of a contract or mandate, without coming under the direct authority of that party. (Protection of Personal Information Act, 2013 s1)	*Called a responsible party*, meaning a public or private body or any other person which/who, alone or in conjunction with others, determines the purpose of and means for processing personal information (Protection of Personal Information Act, 2013 s1) An Information Officer, as per the Protection of Personal Information Act, 2013, has responsibilities that include: Encouraging compliance by the body with the conditions for the lawful processing of personal information; dealing with requests made to the body pursuant to this Act; working with the Regulator in relation to investigations conducted pursuant to Chapter 6 in relation to the body; ensuring compliance by the body with the provisions of this Act. Other duties as may be prescribed. Officers must take up their duties in terms of this Act only if the responsible party has registered them with the Regulator. (Protection of Personal Information Act, 2013 s55)	*Called an Information Officer* of, or in relation to a – (a) (a) public body means an information officer or deputy information officer as contemplated in terms of s 1 or 17; or (b) private body means the head of a private body as contemplated in s 1 of the Promotion of Access to Information Act (Protection of Personal Information Act, 2013 s1)
*Tanzania*	Means the subject of personal data which are processed under this Act (The Personal Data Protection Act, s3)	Means a natural person, legal person or public body which processes personal data for and on behalf of the controller and under the data controller’s instruction, except for the persons who, under the direct authority of the controller, are authorized to process the data and it includes his representative (The Personal Data Protection Act, s3)	Means a natural person, legal person or public body which alone or jointly with others determines the purpose and means of processing of personal data; and where the purpose and means of processing are determined by law, the ‘data controller’ is the natural person, legal person, or public body designated as such by that law. This definition also includes the representative of the data controller. (The Personal Data Protection Act, s3)	Means an individual appointed by the data controller or data processor charged with ensuring compliance with the obligations provided for in this Act (The Personal Data Protection Act, s3)
*Uganda*	Means an individual from whom or in respect of whom personal information has been requested, collected, collated, processed or stored. (The Data Protection and Privacy Act, 2019 s2)	Means the person other than an employee of the data controller who processes the data on behalf of the data controller. (The Data Protection and Privacy Act, 2019 s2)	Means a person who alone, jointly with other persons or in common with other persons or as a statutory duty determines the purposes for and the manner in which personal data is processed or is to be processed (The Data Protection and Privacy Act, 2019 s2)	Not defined in the Act but provided for under s6 of the Act. Responsible for ensuring compliance with the Act. (The Data Protection and Privacy Act, 2019 s6)
*Zimbabwe*	An individual who is an identifiable person and the subject of data. (Data Protection Act 2021 [*Chapter 11:12*] s3)	A natural person or legal person, who processes data for and on behalf of the controller and under the controller’s instruction, except for the persons who, under the direct employment or similar authority of the controller are authorized to process the data. (Data Protection Act 2021 [*Chapter 11:12*] s3)	Any natural person or legal person who is licensable by the Authority; includes public bodies and any other person who determines the purpose and means of processing data. (Data Protection Act 2021 [*Chapter 11:12*] s3)	Any individual appointed by the data controller and is charged with ensuring, in an independent manner, compliance with the obligations provided for in this Act. (Data Protection Act 2021 [*Chapter 11:12*] s3)

The nomenclature adopted in South Africa differs, although the meaning and roles remain roughly equivalent. In POPIA, the data controller is known as the ‘responsible party’. A data processor is not directly employed by the data controller, but is processing the personal data under the direction of the data controller. In the research context, this may be a consultant. In South Africa, a data processor is known as an operator. A data protection officer (DPO) is a person in an organization who is appointed to advise and promote compliance with the law. In South Africa, this person is known as an Information Officer, and in Botswana the person is called a Data Protection Representative. A DPO is not defined in Nigeria, Malawi, or Rwanda, whereas a DPO is not defined but provided for in Kenya and Uganda.

#### 4. Requirements for the processing of personal data

All countries with data protection acts set out the requirements for the lawful processing of data. As can be seen from Table 4, there are variations on the exact requirements but, typically, they require a lawful basis for the processing of personal data, follow the principles of data minimization, purpose limitation and storage limitations, have requirements on the accuracy of the data and/or data quality, security safeguards, and provide data subjects with rights.

**Table 4:** Conditions for the lawful processing of personal data

**Country**	**Conditions for the lawful processing of personal data**
*Botswana*	Lawfulness and fairness (section 14(a)) Adequacy (section 14(b)) Accuracy and completeness (section 14(c)) Purpose limitation (section 14(d)) Security (section 14(f)) Completeness and correction (section 14(g)) Storage limitation (section 14(h)) Good practice (section 14(i)) Processing limitation (sections 14(e) and 15)
*Kenya*	Processed in accordance with the right to privacy of the data subject (section 25(a)) Processed lawfully, fairly and in a transparent manner in relation to any data subject (section 25(b)) Collected for explicit, specified and legitimate purposes and not further processed in a manner incompatible with those purposes (section 25(c)) Adequate, relevant, and limited to what is necessary in relation to the purposes for which it is processed (section 25(d)) Collected only where a valid explanation is provided whenever information relating to family or private affairs is required (section 25(e)) Accurate and, where necessary, kept up to date, with every reasonable step being taken to ensure that any inaccurate personal data is erased or rectified without delay (section 25(f)) Kept in a form which identifies the data subjects for no longer than is necessary for the purposes which it was collected (section 25(g)) Not transferred outside Kenya, unless there is proof of adequate data protection safeguards or consent from the data subject (section 25(h))
*Malawi*	Lawfulness of data processing (section 8) Provision of information (section 15) Purpose limitation (section 9) Data minimization (section 10) Storage limitation (section 12) Accuracy (section 11) Data integrity and data confidentiality (section 13)
*Nigeria*	Lawfulness, fairness and transparency (section 24(1)(a)) Purpose limitation (section 24(1)(b)) Adequacy (section 24(1)(c)) Storage limitation (section 24(1)(d)) Accuracy (section 24(1)(e)) Security safeguard (section 24(1)(f)) Security, integrity and confidentiality (section 24(2)) Accountability and duty of care (section 24(3))
*Rwanda*	Personal data are processed lawfully, fairly and in a transparent manner (Article 37 (1°)) Personal data are collected for explicit, specified and legitimate purposes and not further processed in a manner incompatible with those purposes (Article 37 (2°)) Personal data are related to the purposes for which their processing was requested (Article 37 (3°)) Personal data are accurate and, where necessary, kept up to date, with every reasonable step being taken to ensure that any inaccurate personal data are erased or rectified without delay (Article 37 (4°)) Personal data are kept in a form which permits identification of data subjects for no longer than is necessary for the purposes for which the personal data are processed (Article 37 (5°)) Personal data are processed in compliance with the rights of data subjects (Article 37 (6°))
*South Africa*	Purpose specification (sections 13, 14) Processing limitation (sections 9, 10, 11, 12) Information quality (section 16) Further processing limitation (section 15) Openness (sections 17, 18) Data subject participation (section 23 [access], 24 [correction]) Security safeguards (sections 19, 20, 21, 22) Accountability (section 8) Correction of personal information (section 24) Lawfulness (section 9(a)) Privacy (section 9(b)) Minimality (section 10) Storage limitation (section 14)
*Tanzania*	Lawfulness, fairness, and transparency (section 5(a)) Purpose limitation (section 5(b)) Data minimization (section 5(c)) Accuracy (section 5(d)) Storage limitation (section 5(e)) Data subject rights (section 5(f)) Integrity (section 5(g)) and confidentiality (section 5(g) or 5(h))
*Uganda*	Accountability (section 3 (1)(a)) Collect and process data fairly and lawfully (section 3 (1)(b)) Data minimization (sections 3 (1)(c) and 14) Storage limitation (sections 3 (1)(d) and 18) Data quality (sections 3 (1)(e) and 15) Openness (section 3 (1)(f)) Observe security safeguards in respect of the data (section 3 (1)(g)) Consent (section 7) Privacy (section 10) Purpose limitation (section 12) Accuracy (section 16) Security (sections 20, 21) Cross-border transfer limitation (section 19)
*Zimbabwe*	Data quality (section 7) Accessibility (section 7(2)) Lawfulness and fairness (section 8) and transparency (section 13(b)) Consent (sections 10 [non-sensitive] and 11 [sensitive] and 12 [genetic data, biometric sensitive data and health data]) Privacy (section 13(a)) Purpose limitation (sections 9 and 13(c)) Data minimization (section 8) Accuracy (sections 7(b) and 13(f)) Storage limitation (section 7(c)) Disclosure (sections 15 and 16) Security (section 18) Accountability (section 24)

#### 5. Rights of data subjects

All countries with data protection acts provide certain rights for data subjects. As illustrated in Table 5, there are variations in the exact rights that are provided for. All countries provide a right to access and a right to information. In Uganda, the right to information is not explicitly provided for, but the data subject must still be provided with certain information. All countries, with the exception of Botswana and Kenya, provide for rights in relation to automated decision-making and profiling. All countries except for Botswana provide for a right to either object to or to prevent processing.

**Table 5:** Rights of data subjects

	**Botswana**	**Kenya**	**Malawi**	**Nigeria**	**Rwanda**	**South Africa**	**Tanzania**	**Uganda**	**Zimbabwe**
Information	X	X	X	X	X	X	X	X	X
Prevent processing			X		X	X	X	X	
Prevent processing for direct marketing purposes			X			X	X	X	
Relation to automated decision-making and profiling		X	X	X	X	X	X	X	X
Compensation							X	X	
Rectification, blocking, erasure and destruction of personal data	X		X			X	X	X	X
Access	X	X	X	X		X		X	X
Correction	X		X	X		X			X
Withdraw consent			X	X					X
Object		X	X	X	X	X			X
Data portability			X	X	X				
Rectification	X	X	X	X	X				X
Erasure	X	X	X	X	X	X			X
Restriction		X	X	X	X				
Lodge a complaint	X		X	X				X	
Designate an heir					X				

Some countries provide for certain exceptions to these rights for research. The right to information and the right to access can be derogated from in Botswana if the processing is for research. In Rwanda, an exception is provided for the right to erasure of personal data where the processing is for scientific research. In Zimbabwe and South Africa, the right to information can be exempted from if the personal data has not been collected directly from the data subject and the processing is for research purposes. In Malawi, sensitive personal data may be processed for the purpose of research.

There are no exceptions to data subject rights for research in Ghana, Uganda, Tanzania, and Kenya.

#### 6. Grounds for the cross-border transfer of data for research

Generally, the cross-border transfer of data is not defined in the statues, except in two countries. In Botswana, cross-border flow is defined as ‘the international flow of personal data that can be transmitted by electronic or other forms of transmission, including by satellite’ (section 2 of the Data Protection Act). In Tanzania, cross-border data flow is defined as ‘any international cross-border flows of personal data by means of electronic transmission or other means’.

Ghana has no specific provisions to be met when transferring personal data across borders. Therefore, a researcher who wants to transfer personal data outside Ghana must comply with the general principles and regulations in the Data Protection Act. Therefore, Ghana will not be included in the analysis in the following discussion.

For all other countries, there must be a basis on which to transfer personal data across borders. These conditions must be met in addition to the general requirements set out in the respective legislation, including a lawful basis for processing personal data and processing special personal data. The grounds for transfer can be broadly grouped into (1) adequacy and (2) grounds other than adequacy. We now consider each of these in turn.

##### i. Adequacy

Each country provides for transfer based on some form of adequate level of protection (hereinafter referred to as adequacy) in the country to which the data controller is sharing the data. There are considerable differences in how adequacy is determined in each country.

In Tanzania, a transfer of personal data to another country may occur where the country has a legal framework that provides for adequate data protection and if one of the following has been established:

(i) the recipient establishes that the personal data is necessary for the performance of a task carried out in the public interest or for a purpose related to the lawful functions of a data controller (article 31(2)(*a*)) or,
(ii) the recipient establishes the necessity of having the data transferred and there is no reason to assume that the data subject’s legitimate interests might be prejudiced by the transfer or the processing in the recipient country (article 31(2)(*b*)).

Decisions as to the necessity of the transfer must first be made by the data controller and this must be verified by the recipient. The data controller must also ensure that the recipient processes the personal data for the purposes for which it was transferred.

Where a country does not have a relevant legal framework that provides for an adequate level of protection, the Tanzanian legislation provides that a cross-border transfer of data can still take place if an adequate level of protection is ensured in the country of the recipient and the personal data is transferred solely to permit processing authorized by the controller. An assessment of adequacy is made taking into consideration the following: (i) all the circumstances of the relevant personal data transfer; (ii) the nature of the personal data; (iii) the purpose and duration of the proposed processing; (iv) the recipient’s country; (v) the relevant laws in force in the third country; and (vi) professional rules and security measures are complied with in that recipient’s country.

In Botswana, section 48(1) of the Data Protection Act prohibits the transfer of personal data from Botswana to another country unless the country is listed in the Gazette by the Minister publishing it in an Order (section 48(2)). The cross-border flow of personal data can take place to any country listed without the need for further safeguards. For countries not on the list, the cross-border flow of personal data can only take place if the third country to which the data is transferred provides an adequate level of protection (section 49(1)). This assessment is carried out by the Commissioner, who will determine whether the third country to which the data is being transferred has an adequate level of protection (section 49(2)). This assessment depends on the circumstances of each case, with particular consideration being given to: (i) the nature of the data (section 49(2)(*a*)); (ii) the purpose and duration of the proposed processing operation (ie the research) (section 49(2)(*b*)); (iii) the country of origin and country of final destination (section 49(2)(*c*)); (iv) the rule of law, both general and sectoral, in force in the third country (section 49(2)(*d*)); and (v) the professional rules and security safeguards which are complied with in that country (section 49(2)(*e*)).

In Zimbabwe, adequacy is assessed having considered all the circumstances of a data transfer operation. It provides that particular consideration be given to the nature of the data, the purpose and duration of the proposed processing operation, the recipient country, the laws relating to data protection in force in the country, and the professional rules and security measures which are complied with in that country (section 28(2)).

In Nigeria, a transfer based on adequacy can occur when the recipient of the personal data is subject to a law, binding corporate rules (BCR), contractual clauses, code of conduct or certification mechanism that affords an adequate level of protection to personal data in accordance with the Act (section 41(1)). Having selected a mechanism to transfer, the data controller must then assess whether the level of protection afforded by the recipient country is ‘adequate’ for the purposes of this Act (section 41(2)). In considering whether the level of protection is adequate, the data controller or data processor can take into account: (i) the availability of the data subject’s enforceable rights and ability to enforce such rights through administrative and judicial redress; (ii) the availability of any appropriate instrument in place between the Commission and a competent authority in the recipient jurisdiction that guarantees ‘adequate’ data protection; (iii) the access of public authority to personal data; (iv) the existence of an effective data protection law; (v) the existence of an independent and competent data protection or similar supervisory authority; and (vi) the relevant country being bound by international commitments or conventions and by its membership of any multilateral or regional organizations (section 42(2)). Regarding determining the adequacy of the law in the recipient country, the list developed by the National Information Technology Development Agency (NITDA) in the Nigeria Data Protection Regulation 2019 (NDPR) Implementation Framework is applicable. In addition, Nigeria deems any country that has ratified the Malabo Convention as adequate.

Under Malawi’s Data Protection Act, the assessment is similar to Nigeria’s. The Act provides that the recipient of the personal data can be subject to a law, BCR, contractual clauses, code of conduct, or certification mechanism that affords an adequate level of protection with respect to the personal data (section 38(1)). The Authority may also decide whether an international organization or a recipient of personal data outside Malawi provides an adequate level of protection of personal data based on comparable adequacy decision made by a competent data protection authority in another country (section 39(3)). The Authority is responsible for this assessment and will take into consideration: (i) the availability of data subject rights and the availability of mechanisms for data subjects to enforce their rights through administrative or judicial processes (section 39(2)(*a*)); (ii) respect for the rule of law and human rights and freedoms by the country (section 39(2)(*b*)); (iii) the existence of a legally binding instrument between the Authority and the relevant public authority in the country, addressing elements of adequacy of data protection (section 39(2)(*c*)); (iv) the prevailing policy on access to personal data by a public authority in the country (section 39(2)(*d*)); (v) the existence of an effective data protection law in the country (section 39(2)(*e*)); (vi) the existence of a functionally independent and competent data protection or similar supervisory authority with adequate enforcement powers (section 39(2)(*f*)); and (vii) international commitment and convention binding on the country, including its membership in a relevant multilateral or regional organization (section 39(2)(*g*)).

The Act further provides that the Minister may give notice in the Gazette of any country, region, or specified sector in a country or a standard personal data protection contractual clause that it has determined as affording an adequate level of protection (section 39(5)). The Authority may approve BCR, codes of conduct, or certification mechanisms proposed to it by a data controller, where the Authority determines that they have adequate protection (section 40). The Authority may also decide whether an international organization or a recipient of personal data outside Malawi provides an adequate level of protection of personal data based on comparable adequacy decision made by a competent data protection authority in another country (section 39(3)).

South Africa, Uganda, and Rwanda all provide for transfer based on adequacy but have less detailed provisions. In South Africa, POPIA states that there must be an adequate level of protection in the form of a law, BCR, or a binding agreement. In Uganda, the legislation states that the country where the data is processed or stored must have adequate measures in place for the protection of personal data, at least equivalent to the protection provided for by the Act. Rwanda requires a data controller or processor to obtain authorization from the supervisory authority after providing proof that the outside country has appropriate provisions (article 48(1)).

In Kenya, there are eight legal bases on which a transfer can occur, including adequacy. However, the transfer of sensitive personal data is permissible only if the data subject has consented to the transfer and there are appropriate safeguards (section 49(1)). If sensitive personal data can be shared on this ground, the Data Commissioner may request a demonstration of the effectiveness of the security safeguards or the existence of compelling legitimate interests (section 49(2)). To protect the rights and fundamental freedoms of data subjects, the Data Commissioner may prohibit, suspend or subject the transfers to such conditions as may be determined (section 49(3)).

##### ii. Grounds other than adequacy

Outside of adequacy, the other grounds on which personal data can possibly be transferred are listed in Table 6. Typically, the grounds are: (i) consent; (ii) the transfer is necessary for the performance of a contract between the data subject and the data controller; (iii) vital interests; (iv) legitimate interests; (v) adequate safeguards; (vi) public interest grounds; (vii) transfer from a public register; (viii) benefit to the data subject; and (ix) impossibility to obtain consent.

**Table 6:** Grounds for cross-border flow of data outside of adequacy

	**Consent**	**Contract**	**Performance of a contract**	**Vital interests**	**Legitimate interests**	**Public register**	**Adequate safe-guards**	**Public interest**	**Benefit**
*Botswana*	X (section 49(5))	X (section 49(5)(a))	X (section 49(5)(a))	X (section 49(5)(d))		X (section 49(5)(e))	X (section 49(6))	X (section 49(5)(c))	
*Kenya*	X (section 49 (1)	X (section 48(1)(c)(i))							
*Malawi*	X (section 39(4)(a))	X (sections 39(4)(b) and (c))	X (sections 39(4)(b) and (c))						X (section 39(4)(d))
*Nigeria*	X (section 43(1)(a))	X (section 43(1)(b))	X (section 43(1)(b))	X (section 43(1)(f))				X (section 43(1)(d))	X (section 43(1)(c))
*Rwanda*	X (Article 48 2°)	X (Article 48 3°(a))	X (Article 48 3°(b))	X (Article 48 3°(e))	X (Article 48 3°(f))		X (Article 48 1°)	X (Article 48 3°(c))	
*South Africa*	X (section 72(b))	X (section 72(c) and (d))	X (section 72(c) and (d))						X (section 72(e))
*Tanzania*	X (section 32(4)(a))	X (section 32(4)(b))	X (section 32(4)(b))		X (section 32(4)(e))		X (section 32(5))	X (section 32(4)(d))	
*Uganda*	X (section 19(b))						X (section 19 (a))		
*Zimbabwe*	X (section 29(1)(a))	X (section 29(1)(b))	X (section 29(1)(b))	X (section 29 (e))	X (section 29(1)(f))	X (section 29(1)(f))		X (section 29(1)(d))	

In addition to these grounds, Rwanda provides additional grounds that include: (x) the transfer is necessary to protect the interest of a data subject or of another person where the data subject is physically or legally unable to give their consent (article 48(3)(*e*)); and (xi) the transfer is for the performance of international instruments ratified by Rwanda (article 48(3)(*g*)). Furthermore, the supervisory authority can decide on additional grounds for sharing or transferring personal data to a third party outside Rwanda.

##### iii. Other requirements

Some countries have additional requirements and provisions for the cross-border sharing of data. Zimbabwe provides that the Authority can lay down categories of processing operations and the circumstances in which data transfer to countries outside Zimbabwe is authorized (section 28(3) of the Data Protection Act). In Rwanda, if a data controller or data processor authorizes a person to access personal data and share or transfer the data to a third party outside Rwanda, they must enter into a written contract with such a person. This contract must set out the respective roles and responsibilities of each party to ensure compliance with the law (article 49). The Supervisory Authority may, by a regulation, determine the form of the contract to be used for transfers of personal data outside Rwanda (article 49). The Supervisory Authority may require the data controller or the data processor to demonstrate their compliance with the provisions of this Article and, in particular, with personal data security safeguards and interests as specified in Article 48(3)(*f*). In addition, the Supervisory Authority may prohibit or suspend the transfer of personal data outside Rwanda in order to protect the personal rights and freedoms of the data subject (article 49). Furthermore, the storage of personal data outside Rwanda is permitted only if the data controller or the data processor holds a valid registration certificate authorizing them to store personal data outside Rwanda, and which is issued by the Supervisory Authority (article 50).

Tanzania requires that, in addition to a legal basis for cross-border data sharing, section 20 of the Personal Data Protection (Personal Data Collection and Processing) Regulations 2023, provides that a data controller or data processor who intends to transfer personal data outside the country apply for a permit using Form No. 7 set out in the First Schedule to the Regulations. The application must include the following information: (i) particulars of the applicant; (ii) particulars of the recipient; (iii) particulars of the data subject; (iv) the type of personal data to be transferred; (v) the purpose and necessity of transferring personal data; (vi) details of the security of personal data in the country of the recipient; (vii) consent of the data subject; (viii) date and time of sending personal data; and (ix) any other information as may be required by the Commission. In addition, at the time of application, proof must be submitted that the country receiving the personal data has ratified an international agreement that specify details on the protection of personal data; there is an agreement between the Republic and the country receiving the personal data regarding the protection of personal data, or there is a contractual agreement between the person requesting the personal data and the recipient of the personal data who is outside the country. The Commission must consider an application within 14 days, after which time it can reject or approve a permit. An application may be rejected for the following reasons: (i) the transfer of personal data endangers national security; (ii) the Commission is satisfied that there is inadequate protection of personal data in the country of the recipient; (iii) other written laws restrict the transfer of personal data; (iv) the application for the permit to transfer personal data does not meet the requirements of Regulation 20; and (v) other reasonable grounds which the Commission may deem necessary for the public interest. Finally, the permit is issued subject to the following conditions: (i) the personal data must be transferred to the recipient authorized in the permit; (ii) the personal data transferred must be processed for the intended purpose only; (iii) the personal data must not be disclosed or transferred to another recipient without the approval of the Commission; and (iv) the processing of personal data outside the country must not violate the laws of the country.

#### 7. Additional requirements from ethics frameworks

In addition to the requirements as set out in the applicable data protection legislation, additional requirements are set out in national research ethics legislation and/or guidance for the cross-border sharing of data for research. This is in addition to the general research ethics requirements, such as informed consent, research ethics committee oversight, and other requirements. Table 7 sets out the relevant national research ethics legislation and guidance in each country. Table 8 sets out the additional requirements imposed by national research ethics requirements for cross-border data sharing for research.

**Table 7:** Applicable national legislation and ethical guidance

**Country**	**Applicable national legislation & ethical guidance**
*Botswana*	Constitution of the Republic of Botswana, 1966 Public Health Act (Chapter 63:01)
*Cameroon*	Law No 2022/008 of 27 April 2022 Relating to Medical Research Involving Human Subjects
*Ghana*	Public Health Act, 2012 (Act No. 851) The Council for Scientific and Industrial Research (CSIR) Act 1996 The Standard Operating Procedures of CSIR Institutional Review Board
*Kenya*	Science, Technology and Innovation Policy 2020–2030 (September 2020) National Guidelines for Ethical Conduct of Biomedical Research Involving Human Participants in Kenya (January 2020) National Guidelines for Registration, Licensing, and Regulation of Researchers in Kenya (July 2022) National Guidelines for Registration of Research Institutions in Kenya (January 2020) Guidelines for Accreditation of Institutional Ethics Review Committees in Kenya (October 2017) Ethical Guidelines for Public Health Emergencies in the Response to COVID-19 Pandemic in Kenya (December 2020)
*Malawi*	Constitution of Malawi, 1994 Public Health Act, 1948 Pharmacy and Medicines Regulatory Authority Act no. 9 of 2019 The National Health Research Agenda, 2012 Policy Requirements, Procedures and Guidelines for the Conduct and Review of Human Genetic Research in Malawi, 2012 National Policy Measures and Requirements for the Improvement of Health Research Co-ordination in Malawi, 2012. This was published by the National Commission for Science and Technology and relates to sections 18 and 48 of Malawi’s Science and Technology Act 16 of 2003.
*Nigeria*	The Constitution of the Federal Republic of Nigeria, 1999 The National Health Act, 2014 National Code of Health Research Ethics 2007
*Rwanda*	Ministerial Instructions No 003/2010 of 09/12/2010 Rules and Regulations for Research Activities (In accordance with the Ministerial Instructions No 003/2010 of 09/12/2010 published in the official Gazette of the Republic of Rwanda of 24/12/2010 Regulating research activities in Rwanda) Health Sector Research Policy, 2012 Law of Establishing the National Cyber Security Authority and Determining its Mission, Organization and Functioning, 2017 Health Sector Policy, 2015 Regulations governing the conduct and inspection of Clinical Trials in Rwanda
*South Africa*	Department of Health (2020): South African Good Clinical Practice: Clinical Trial Guidelines, 3^rd^ edition The South African Medical Research Council (2018): Guidelines on the Responsible Conduct of Research National Health Act: Material Transfer Agreement of Human Biological Materials (SA MTA) of 20 July 2018 Department of Health (2015): Ethics in Health Research: Principles, Processes and Structures Guidelines, 2^nd^ edition Regulations Relating to Research with Human Participants GN R719 GG 38000 of 19 September 2014 Regulations relating to the Import and Export of Human Tissue, Blood, Blood Products, Cultured Cells, Stem Cells, Embryos, Foetal Tissue, Zygotes and Gametes GN R181 GG 35099 of 2 March 2012
*Tanzania*	The Constitution of the United Republic of Tanzania Tanzania National Scientific Research Council Act, 1968 Tanzania National Scientific Research Council (Amendment) Act, 1981 Tanzania Commission for Science and Technology Act, 1986 Human DNA Regulations Act, 2009 Tanzania Food, Drugs and Cosmetics Act, 2003 Guidelines of Ethics for Health Research In Tanzania, 2009
*The Gambia*	The National Health Policy 2021–2030 The National Health Laboratory Services Strategic Plan 2021–2025 The Gambia ICT4D Policy framework The National Science, Technology and Innovation Policy (NSTIP) (2013–2022)
*Uganda*	The National ICT Policy, 2014 The Uganda Health Research Organization Act, 2009 National Guidelines for Research Involving Humans as Research Participants, 2014 Guidelines on Good Clinical Practice in the Conduct of Clinical Trials Involving Human Participants, 2019 The Public Health Act, 1935 The Access to Information Act, 2005
*Zimbabwe*	Constitution of Zimbabwe Act No. 20 of 2013 Research (Constitution of the National Public Health Institute) Regulations, 2020 Research Act [Chapter 10:22]

**Table 8:** Additional ethical rules on cross-border flow of data

**Country**	**Additional ethical rules on cross-border flow of data**
*Botswana*	No extra provisions on cross-border sharing of data.
*Cameroon*	Non-genetic health-related personal data may be disclosed abroad for research purposes if: • the data subject consents; • there is a written data-sharing agreement; and • a national investigator is involved in the research project in question. Genetic data may be transferred abroad for research purposes if: • the data subject has given his or her free, informed and written consent; • the body in charge of ethics establishes that the research cannot be conducted in Cameroon; and • a national investigator is involved in the research project in question.
*Ghana*	No extra provisions on cross-border sharing of data.
*Kenya*	International collaborative research involving collaborative research requires the involvement of a Kenyan PI.
*Malawi*	Transfer of genetic material (locally or nationally) can take place only if: • the researcher and the other research group are collaborating on a research study that has been approved by the National Health Sciences Research Committee (NHSRC); • genetic material and information is provided in a form that ensures that participants cannot be identified; and • the research group ensures that privacy and confidentiality are not compromised in holding the material and information. Cross-border movement of genetic material is not permitted unless: • There is a justifiable reason to do so. • The NHRSRC has approved and reviewed the study. • The MTA for the cross-border movement has been reviewed and signed by the NHSRC. To transfer genetic material, the NHSRC must approve the research study.
*Nigeria*	No extra provisions on cross-border sharing of data.
*Rwanda*	No extra provisions on cross-border sharing of data.
*South Africa*	National Material Transfer Agreement (SA MTA) requires that a relevant Human Research Ethics Committee (HREC) first approve the MTA before a transfer of human biological material and its accompanying data can occur.
*Tanzania*	Permission required from the Office of the Regulator of Human DNA Services to send samples for human DNA analysis abroad. National Institute for Medical Research (NIMR) must approve all research that involves foreign researchers or collaborators.
*The Gambia*	No extra provisions on cross-border sharing of data.
*Uganda*	REC must approve any cross-border data sharing. There must be a local PI. There must be a MTA.
Zimbabwe	No extra provisions on cross-border sharing of data.

Six countries (Botswana, Ghana, Kenya, Nigeria, Rwanda, and The Gambia) have no extra requirements outside of the general research ethics requirements that apply to the cross-border sharing of data for research. The remaining six countries have differing requirements that include a material transfer agreement (MTA), designation of a local PI, through to some other official approval being required. South Africa requires a human research ethics committee (NHREC) to approve and sign an MTA. Data is allowed to be shared outside of Malawi only if: (i) there is a justifiable reason to do so; (ii) if the National Health Sciences Research Committee (NHSRC) has reviewed and approved the study; (iii) if the NHSRC has reviewed and approved the MTA; (iv) if the genetic material and information is provided in a form that ensures that participants cannot be identified; and (v) if the research group ensures that privacy and confidentiality are not compromised in holding the material and information. Tanzania requires approval from the National Institute for Medical Research (NIMR) for all research that involves foreign researchers or collaborators, and it is an offence to send samples for human DNA analysis abroad without the permission of the Office of the Regulator of Human DNA Services. The transfer of genetic data outside of Cameroon can take place only if: (i) the data subject has given his or her free, informed, and written consent; (ii) the body in charge of ethics establishes that the research cannot be conducted in Cameroon; and (iii) a national investigator is involved in the research project in question. For other health data, the transfer can occur outside of Cameroon only if: (i) the data subject consents; (ii) there is a written data-sharing agreement; and (iii) a national investigator is involved in the research project in question. Uganda requires a research ethics committee (REC) to approve any cross-border data sharing, the researcher must be a local PI, and an MTA must be signed. Finally, international collaborative research can occur in Kenya only if a Kenyan PI is involved.

## III. DISCUSSION

A reading of the cross-country reports demonstrates that there are considerable similarities between countries and that these similarities extend to similarities with the GDPR, Convention 108, and other international standards in data protection. They include similarities in the lawful bases for processing of data, the application of data protection law, key individuals under data protection law, and the rights of data subjects. While there are similarities in the grounds for the lawful basis for transfer of health data outside of each country, with most countries adopting some, if not all, of the lawful grounds provided for under the GDPR, careful analysis demonstrates that any attempts at a harmonized approach to cross border data sharing has failed on the continent, a finding that is unsurprising considering that out of the countries surveyed, only five countries have signed the Malabo Convention and only two countries have ratified it. It is also clear that guidance from national supervisory authorities are required to guide the application of the national legislation to research. Thus, while health data that includes genetic data generally can be shared across borders for research, it currently is a complex, fragmented, and uncertain landscape.

### III.A. Meaning of Cross-Border Data-Sharing

First, what is considered to be cross-border data sharing is not always immediately clear, particularly for countries where cross-border data sharing is not defined. This lack of definition makes it complex in an area of data driven research and the increasing use of clouds to store data. The guides do make some attempts to acknowledge and address this point. The country guides state that while the cross-border data-sharing provisions apply when personal data is sent to a data controller in another country, there are other times when it could apply. For example, it could also apply where a researcher outside of the country accesses personal information in the country, or when data is put onto a cloud where the server is not hosted in the country.

It is thus clear that physically transferring data from one country to another does fall under the cross-border data provisions. What is less clear is whether if a researcher is in Country A and is accessing data from Country B through a mechanism that enables them to view the data only and not download, whether that amounts to cross border data sharing. It is also not clear whether if data is held on a server outside of a country, whether that amounts to cross border data sharing. Unlike in the EU where the European Data Protection Board (EDPB) has issued considerable guidance in this space, no supervisory authority from the countries surveyed had issued any guidance on this point. Considering that researchers in many African countries may struggle to secure the necessary infrastructure and resources to store health data in country, this is an issue that needs discussion and clarification as a priority.

### III.B. Application of Data Protection Legislation

A second issue is that there is uncertainty about the application of the relevant data protection legislation. In most countries, as under the GDPR, data protection legislation would apply to the processing of *personal* data. Unlike the GDPR, where the EDPB has issued guidance on a test for anonymization, such guidance is lacking across the countries surveyed and they can only rely on the definition provided on the definition of personal data. Thus, when making an assessment about whether the data is personal, it is unclear whether researchers must consider whether anyone in the world can identify a data subject or whether the individual to whom the researcher is sending the data can identify the data subject.^12^ In the absence of direction from all supervisory authorities on this point, the country guides state that it is for the data controller to decide. To guide researchers in making this decision – and to err on the side of caution – the country guides provide the following general points, many of which come from guidance related to anonymization under the GDPR:

- An assessment must be made on a case-by-case basis, considering the particular context.
- The anonymization must be irreversible.
- The assessment is made on the current state of the art. As technology progresses, data that was once deemed anonymous may become personal data and therefore fall under data protection laws.
- Consider all means of identification that a person could use, for example, available datasets.
- Consider objective factors that are *reasonably likely* to be used, such as technology, resources, and time, to identify.
- You may wish to follow the GDPR test: if an individual cannot be singled out, or identifiers cannot be linked to make a person identifiable, or if it is impossible to infer a link between two pieces of information in a dataset, then the data is anonymous.
- Genetic data is considered sensitive personal data. It not only falls under the data protection laws but has a higher level of protection.

This assessment is even more challenging in the light of the ongoing debate about whether genomic datasets can ever be truly anonymized, particularly as genetic data is an identifier.^13^ Guidance on these issues is needed, particularly for data-driven researchers where data is being linked. Researchers must therefore navigate each regulatory framework to determine the ground under which they can share data. This can be challenging for multi-site and multi-jurisdictional research and needing to identify grounds that may differ in each jurisdiction is likely to be a disincentive for international collaborative research. Considering the considering legal implications of data being considered personal data, guidance is critically needed from supervisory authorities on this point.

### III.C. Grounds for Cross Border Data Sharing

Turning to the grounds under which personal data can be transferred across borders for research, across the countries surveyed, the grounds are quite similar to that provided by the GDPR. Despite this, there is a lack of harmonization as there is no one ground that could apply to all countries to enable the legal transfer of health data across borders.

Consent and adequacy are the most common grounds provided for in the legislation that we studied. Consent as a ground for transfer is provided for in each country and, notably, is the only ground under which health data can be transferred in Kenya. For data-driven research methods, obtaining specific consent for a specific data transfer from thousands of participants in many countries may not be feasible. A solution to this, however, could be dynamic consent. Dynamic consent is a consent model that uses digital technologies, such as apps, to enable participants to consent to data use, update their consenting preferences, and received ongoing information about their data use.^14^ In the context of cross-border transfers of data that require specific consent to that specific data transfer, dynamic consent would enable the ease of contact with participants to obtain their consent to the data transfer. While dynamic consent could be a solution, its implementation requires investment into infrastructure, including software and security systems, but also the availability of mobile phones, internet connections, and digital literacy. Dynamic consent is also suitable only for prospective data collection.

Adequacy is also a ground under which health data can be shared for research in the majority of countries surveyed, but the test for adequacy differs in each country. Researchers face a legal maze where the criteria for transferring personal data based on adequacy vary significantly from one country to another. Some jurisdictions have developed approved lists of countries deemed adequate, while others require a national authority’s determination or delegate this responsibility to the data controller, sometimes necessitating further validation by supervisory authorities. The failure to secure a harmonized approach to the determination of adequacy will likely result in a lack of coherence in the application of adequacy, which will negatively impact the cross-border sharing of data for researchers. This variability underscores the challenges and uncertainties relating to data sharing across African borders. This compels researchers to meticulously navigate each country’s data protection legislation and, in particular, its cross-border data sharing provisions.

The concept of an African single data market stands out as a strategic solution to these challenges. Supported by the African Union’s initiatives such as the *Digital Transformation Strategy for Africa*^15^ and the *AU Data Policy Framework*,^16^ this idea aims to facilitate seamless data exchange across the continent through legal harmonization. Such harmonization would involve integrating markets, standardizing online payment systems, harmonizing taxation, and easing cross-border trade, thereby simplifying the legal and operational landscape for data sharing.

The *African Continental Free Trade Agreement* (AfCFTA)^17^ presents a unique opportunity to formalize data governance across Africa. The anticipated digital trade protocol to AfCFTA promises to address key areas such as data transfers, data protection, and cybersecurity, establishing a uniform legal framework to govern the exchange of personal data, including health data. Key elements of the draft version of the protocol that is publicly accessible are as follows:^18^

- *Protection of personal data* (Article 21): This provision mandates each State Party to establish a legal framework protecting personal data, which includes health data as a subset. The protocol encourages taking into account principles and guidelines from regional and international bodies. It highlights transparency in how protections are provided, how remedies can be sought, and ensuring compliance for entities involved in digital trade.
- *Cross-border data transfers* (Article 20): The protocol allows the transfer of data, including personal data, by electronic means across borders for digital trade, subject to specific provisions in an annex. This section introduces restrictions to protect legitimate public policy objectives or essential security interests, indicating sensitivity around data types such as health information.
- *Cooperation on data innovation* (Article 23): State Parties are encouraged to collaborate on data-sharing projects using regulatory sandboxes. This provision could encompass sharing of health data for research or innovation while considering protections and data-sharing frameworks that maintain privacy and security.
- *Establishment of national data protection authorities*: Article 21 emphasizes the importance of establishing dedicated bodies to enforce data protection laws, which would be key in managing health data protection.

The digital trade protocol under AfCFTA will mark a significant step forward for health data sharing across Africa. By providing a harmonized legal framework, the protocol will address longstanding challenges in cross-border data governance, offering a more cohesive and secure approach to handling sensitive health data. With provisions that emphasize data protection, facilitate cross-border data transfers, and encourage innovation through regulatory sandboxes, the protocol will lay a strong foundation for multi-country health research, public health initiatives, and advancements in medical innovation. Additionally, the establishment of national data protection authorities will ensure that personal and health data remain safeguarded, fostering greater trust in digital health projects and encouraging collaboration among African nations.

In addition to the broad framework that will be established by the digital trade protocol, we also propose a country-level strategy to facilitate cross-border data sharing. This strategy is based on the practice currently used in Nigeria, where any country that ratifies the Malabo Convention is deemed to provide adequate protection. This is a critical point because the Malabo Convention already incorporates stringent data protection standards, which the draft protocol builds upon. By leveraging this existing regional instrument, countries can quickly establish trust and enable the secure flow of data. This approach not only simplifies the process but also promotes legal certainty and mutual recognition among African states.

Thus, by aligning with the Malabo Convention, African countries can form an African ‘data corridor’^19^—a network of nations that mutually recognize each other’s data protection regimes as providing adequate protection. Such a corridor would be especially beneficial for the exchange of health data. It would allow institutions to collaborate more effectively on critical public health issues, genomic research, and health innovations while ensuring compliance with data protection laws. This ‘data corridor’ would act as a catalyst for health research, enabling rapid sharing of medical information, outbreak monitoring, and the development of new treatments across the continent.

## IV. CONCLUSIONS

The burgeoning landscape of data protection legislation across Africa, aimed at bolstering the privacy and security of personal data, presents a nuanced challenge for multi-site, data-driven research. As the legal landscape evolves, it creates a mosaic of legal requirements that govern the access to and cross-border transfer of personal data for research purposes. Our comprehensive analysis of data protection frameworks in 12 African countries reveals a fragmented legal approach to cross-border data sharing. Despite the presence of common principles such as adequacy for data transfer, variances in national legislation necessitate that researchers meticulously assess and adapt to each country’s specific legal mandates. This environment demands strategic navigation through diverse legal terrains, emphasizing the need for a harmonized approach to facilitate seamless and responsible data sharing across the continent. This will advance research initiatives while upholding the rights and protections of data subjects.

## Supplementary Material

Template_cross_border_data_sharing_lsaf002

## Data Availability

All materials are available in the appendix.

## Footnotes

1 Byrd JB et al., *Responsible, practical genomic data sharing that accelerates research.* 21 Nat. Rev. Genet. 1–15 (2020); Cook-Deegan R, Ankeny RA, Maxson Jones K. *Sharing Data to Build a Medical Information Commons: From Bermuda to the Global Alliance*. 18. Annu. Rev. Genomics  Hum  Genet. 389–415 (2017); Staunton C, de Vries J. *The governance of genomic biobank research in Africa: reframing the regulatory tilt*. 7 J. Law  Biosci. 1–20 (2020).

2 Sheehan M. *Can Broad Consent be Informed Consent?* 4. Public  Health  Ethics. 226–35 (2011); Tindana P, de Vries J. *Broad Consent for Genomic Research and Biobanking: Perspectives from Low- and Middle-Income Countries*. 17. Annu. Rev. Genomics  Hum. Genet. 375–93 (2016).

3 Staunton C et al. *Safeguarding the future of genomic research in South Africa: Broad consent and the Protection of Personal Information Act No. 4 of 2013.* 109 S. Afr. Med. J. 468 (2019); Thaldar DW, Townsend BA. *Genomic research and privacy: A response to Staunton et al*. 110 Samj, S. Afr. Med. J. 172–174 (2020) .

4 Kaye J et al., *Dynamic consent: a patient interface for twenty-first century research networks*. 23 Eur. J. Hum. Genet. 141–6 (2015); Budin-Ljøsne I et al. *Dynamic Consent: a potential solution to some of the challenges of modern biomedical research*. 18 Bmc. Med. Ethics. 4 (2017).

5 Teare HJA, Prictor M, Kaye J. *Reflections on dynamic consent in biomedical research: the story so far*, 29 Eur. J. Hum. Genet. 649–56 (2021); Mascalzoni D et al., *Ten years of dynamic consent in the CHRIS study: informed consent as a dynamic process*, 30 Eur. J. Hum. Genet. 1391–7 (2022).

6 Forzano F, Genuardi M, Moreau Y (On behalf of the European Society of Human Genetics). *ESHG warns against misuses of genetic tests and biobanks for discrimination purposes*, 29 Eur. J. Hum. Genet. 894–896 (2021); Joly Y, Dalpe G. G*enetic discrimination still casts a large shadow in 2022*, 30 Eur. J. Hum. Genet. 1320–2 (2022); Joly Y, Dalpe G. G*enetic discrimination still casts a large shadow in 2022*, 30 Eur. J. Hum. Genet. 1320–2 (2022); de Vries J et al., *Investigating the potential for ethnic group harm in collaborative genomics research in Africa: Is ethnic stigmatisation likely?* 75 Soc. Sci. Med. 1400–7 (2012).

7
*Supra* note 3; Staunton C, Moodley K. *Challenges in biobank governance in Sub-Saharan Africa,* 14 Bmc. Med. Ethics. 35 (2013); Hardy BJ et al., *South Africa: from species cradle to genomic applications,* 9 Nat. Rev. Genet. 19–23 (2008); de Vries J et al., *Ethical issues in human genomics research in developing countries*, 12 Bmc. Med. Ethics. 5 (2011).

8 Kaye J et al., *Data sharing in genomics — re-shaping scientific practice*, 10 Nat. Rev. Genet. 331–5 (2009); Ni Loideain N. *Regulating health research and respecting data protection: a global dialogue*, 10 Int. Data  Priv. Law. 115–6 (2020).

9 African Union Convention on Cyber Security and Personal Data Protection, June 27, 2014, Malabo, Equat. Guinea. https://au.int/en/treaties/african-union-convention-cyber-security-and-personal-data-protection

10 At the time of the study, Cameroon did not have a data protection regulation in place. Law No. 2024/017 on the Protection of Personal Data is now in force.

11 Swales L, Thaldar D, Donnelly DL. *Why research institutions should indemnify researchers against POPIA civil liability*, 118 S. Afr. J. Sci. 22–24 (2022).

12 Thaldar D. *Does data protection law in South Africa apply to pseudonymised data?* 14 Front. Pharmacol. 1–6 (2023)

13 Shabani M, Marelli L. *Re-identifiability of genomic data and the GDPR: Assessing the re-identifiability of genomic data in light of the EU General Data Protection Regulation*, 20 Embo  Rep. (2019)

14
*Supra* note 4, 5; Thaldar DW, Townsend BA. *Exempting Health Research from the Consent Provisions of POPIA*, 24 Potchefstroom  Electron  Law J. 1–32 (2021).

15 African Union. *Digital Transformation Strategy for Africa* (2020). https://au.int/sites/default/files/documents/38507-doc-dts-english.pdf

16 African Union. *AU Data Policy Framework* (2022). https://au.int/sites/default/files/documents/42078-doc-AU-DATA-POLICY-FRAMEWORK-ENG1.pdf

17 African Union. *African Continental Free Trade Agreement* (2018). https://au.int/en/treaties/agreement-establishing-african-continental-free-trade-area

18 African Union. Draft Protocol to the Agreement Establishing the African Continental Free Trade Area on Digital Trade (2024). https://www.bilaterals.org/IMG/pdf/afcfta_digital_trade_protocol_-_9_february_2024_draft.pdf

19 Townsend B. *The lawful sharing of health research data in South Africa and beyond*, 31 Inf. Commun. Technol. Law. 17–34 (2022).

