# Supplementary material for: Cross-border data sharing for research in Africa: an analysis of the data protection and research ethics requirements in 12 jurisdictions
Source: J Law Biosci. 2025 Mar 19;12(1):lsaf002. doi: 10.1093/jlb/lsaf002 (PMC11921095; doi:10.1093/jlb/lsaf002)
Supplement: Template_cross_border_data_sharing_lsaf002 [file template_cross_border_data_sharing_lsaf002.docx]

# Cross border sharing of data in *X*

*Note to RAs: use track changes for all edits/additions. If uncertain of whether to include delete, err on the side of including more than you think as it is quicker to delete rather than go back to the legislation.*

The cross-border sharing of data is governed by several legal and ethical regulations, all of which must be met prior to the sharing of data for research. The (*insert data protection law*) is in force since (*insert year*). It is a general data protection law that applies to the processing (i.e., use) of personal data in all sectors. Therefore, it is not a regulation that was introduced to regulate research. However, as research processes vast quantities of personal data, the (*insert DP law*) applies. It must be remembered that it is only one of several laws that must be met when transferring data for research.

## Health research regulations and cross-border data sharing

In addition to these national laws, there are several international treaties and conventions that have been signed. Of importance in this domain is the African Union Convention on Cyber Security and Personal Data Protection ([the Malabo Convention](https://au.int/sites/default/files/treaties/29560-treaty-0048_-_african_union_convention_on_cyber_security_and_personal_data_protection_e.pdf)) that came into force in June 2023. *(RA to insert whether this country has ratified it*)

The relevant national legislation are:

*RA to list relevant laws, starting with National Health Acts, leaving out the data protection Act.*

These laws set out the legal and ethical requirements that must be met for the conduct of research in *insert country*. Specifically for the cross-border sharing of data,

*RA to write a paragraph answering each of these questions.*

1. *Is international transfer/cross border sharing/any transfer defined?*
2. *Are any approvals required prior to cross border data sharing e.g., REC, a national authority, ministerial?*
3. *Is a legal agreement such as a data transfer agreement required?*
4. *Is there a requirement of a local PI?*
5. *Any other requirements set out to be followed for cross border sharing of data?*

## *RA to insert name of data protection regulation*

In addition to these legal and ethical requirements, the *(insert regulation*) applies to the processing of all personal data, but (*RA to check if there are some special provisions for research. If there are special provisions insert “due to the importance of research, there are special provisions in place for research”. If there are no special provisions insert “unlike most data protection regulations, there are no special provisions in place for research”.)* The conditions set out in the (*insert regulation)* all must be met for research. In addition to this, there are extra conditions that must be met prior to the transfer of personal data across borders. Before considering those, we first turn to discuss some of the techno-legal terms that are used in this Act. Please note that this is not a thorough assessment of the *insert regulation* as it applies to research, but rather an overview of some of the key terms and conditions that must be met in the processing of personal data for research.

### The main actors

|  | **Legal definition** | **Layman explanation** |
| --- | --- | --- |
| **Data subject** |  | This is the person to whom the data relates. |
| **Data controller/Responsible party** |  | This is the person who is deciding on what the data will be used for in research. Legal responsibility falls on the PI *and* on the research institution (as employer). |
| **Data Processor** |  | This is someone that is not directly employed by the data controller but are processing personal data under the direction of the data controller. They may be a consultant, for example. |
| **Information Regulator** |  | This is the independent body established to monitor and enforce compliance with the law |
| **Information Officer/Data Protection Officer** |  | An individual within an organisation appointed to advise and promote compliance with the law. |
|  |  |  |
|  |  |  |

### Categories of data listed in the Act

|  | **Legal definition** | **Layman explanation** |
| --- | --- | --- |
| **Personal data / information** |  | This is data / information about a particular person that can identify him or her. |
| **Sensitive personal data / special personal information** |  | This is a personal data / information about a particular person, such as health data and genetic data, that receives additional legal protection. |
| **Pseudonymised data / information** |  | This is data where the direct identifiers have been removed (e.g., a name) so that it is not possible to identify the person without adding other information. This is often called coded data. Data protection law still applies to pseudonymised data. |
| **Anonymous data** |  | This is data in which it is no longer possible to identify a person from the data. It must not be possible to re-identify the person. Data protection law does not apply to anonymised data. |

Data protection law does not apply to anonymised data. To determine if the data you are sharing is anonymised, it is important to make an assessment, an assessment that a times can be challenging. There is no guidance from the Regulator on this point, but the Data Controller may wish to follow the [guidance](https://ec.europa.eu/justice/article-29/documentation/opinion-recommendation/files/2014/wp216_en.pdf) set out to be followed under the GDPR on how to make an assessment to determine if data is anonymous, including anonymisation techniques.

There is some uncertainty in this area, however, that relates to the perspective from whom we consider if the data is anonymised. On this there are two possibilities:

1. Is there anyone in the world who can identify the data subject from the data? (objective test)
2. Can a specific holder of the data identify the data subject from the data? (subjective test)

The objective test would mean that if Data Controller A sends pseduonymised data (i.e., Data Controller A has the necessary information to identify the people in the dataset) to Controller B, the dataset would not be anonymous.

The subjective test would mean that if Data Controller A sends pseudonymised data (i.e., Data Controller A has the necessary information to identify the people in the dataset) to Controller B, the dataset *may* be anonymous in the hands of Data Controller B. A test would be needed to determine if the dataset is anonymous in the hands of Data Controller B.

In the absence of direction from the regulator on a test, it will be for the Data Controller to decide. In making this decision, there are general points that may be worth keeping in mind (many of which come from guidance related to anonymisation under GDPR):

- An assessment must be made on a case-by-case basis, considering the particular context.
- The anonymisation must be irreversible.
- The assessment is made on the current state of the art. As technology progresses, data that was once deemed to be anonymous may become personal data and thus fall under data protection regulations.
- Take into account all means of identification that could be used by a person e.g., available datasets.
- Consider objective factors that are *reasonably likely* to be used such as technology, resources, time, etc, to identify.
- You may wish to follow the GDPR test that states if an individual cannot be singled out; or identifiers can be linked to make a person identifiable; or it is not possible to infer a link between two pieces of information in a dataset, then the data is anonymous.
- Genetic data is considered sensitive data. It not only falls under the data protection law but has a higher level of protection.
- There is ongoing debate about whether genomic datasets can ever be rendered truly anonymised, particularly as genetic data is an identifier.^[[1]](#footnote-1)^ In considering whether the genomic dataset that you hold can be considered anonymised, remember that context matters i.e., the objective factors surrounding the data.

### Key principles that must be met

*Go through each of these principles in the Act, make sure that they are correct and in line with the principle provided below.*

1. **Lawfulness, fairness, and transparency**: Personal data must be processed lawfully, fairly, and in a transparent manner in relation to the data subject. Lawful means that there must be a legal basis for the processing of the personal data as set on in *insert section*. The processing of sensitive personal data is generally not permitted unless it falls within one of the grounds as set out in *insert section*.
2. **Purpose limitation**: Personal data should be collected for specified, explicit, and legitimate purposes and not further processed in a manner that is incompatible with those purposes. This means that the purpose must be clearly set out.
3. **Data minimisation**: Only the data that is necessary for the specific purpose should be collected and processed. It is essential that only the minimal amount of data that is required to achieve the objectives of the data processing is used.
4. **Accuracy**: Personal data must be accurate and, where necessary, kept up to date. Processes should be in place to ensure that all personal data that is collected is accurate.
5. **Storage limitation**: Personal data should be kept in a form which permits identification of data subjects for no longer than is necessary for the purposes for which the personal data are processed. Once the objective of the processing has been achieved, the data should be deleted. Data that is rendered anonymous does not fall under data protection law. However, to anonymous the data, one must still have a lawful basis to do so.
6. **Integrity and confidentiality**: Personal data should be processed in a manner that ensures appropriate security, including protection against unauthorised or unlawful processing and against accidental loss, destruction or damage, using appropriate technical or organisational measures. It is essential that both organisational and technical measures are put in place to secure the data.
7. **Accountability**: The data controller is responsible for, and must be able to demonstrate compliance with, the principles mentioned above. It is good practice to keep a record of data processing activities, measures introduced to protect the data, as well as any risk assessments made. This can be used to demonstrate compliance in the event of a breach.

*RA to check whether there are special provisions for research for any of these principles e.g., is more information for research made or can any of these principles be exempted from for research*.

### Data subject rights

Data subjects have rights that the Data Controller must protect. These rights are:

1. Right to be informed: the Data Subject has the right be informed about what their personal data will be used for.
2. Right to access: the Data Subject has the right to access personal data that the Data Controller has about them. The Data Controller should have in place a process to facilitate this.
3. Right to rectification: the Data Subject with the right to have inaccurate personal data corrected and incomplete data to be completed.
4. Right to erasure: the Data Subject has the right to request that their data is erased.
5. Right to restriction of processing: the Data Subject can request that the Data Controller stop processing their personal data.
6. Right to data portability: the Data Subject has the right to move their data from one Data Controller to another.
7. Right to object: the Data Subject can object to the processing of their personal data where the lawful basis of processing is not consent.
8. Rights in relation to automated decision making and profiling: the Data Subject has the right to object to a decision based solely on automated processing.

Some rights can be exempted from in the context of research.

*RA to check:*

1. *That the rights above are set out in the legislation.*
2. *Check which rights can be derogated from and in what context and include a sentence or two under each of the rights. An example of what I mean is the following chapter on individual rights under the GDPR. It specifically is on biobanks but you should get an idea of how it can be done for research generally:* [*Individual Rights in Biobank Research Under the GDPR | SpringerLink*](https://link.springer.com/chapter/10.1007/978-3-030-49388-2_6)

### Cross border data sharing

Each of the provisions apply to all research that uses personal data. Data protection law also has additional provisions in place that must be met when personal data is to be transferred outside of the country. These additional provisions are in place to ensure that data subjects continue to be protected once the data leaves the country.

Cross border/transborder data sharing/transfer is not defined. It would apply when the data is being sent to a data controller/responsible party in another country. It *could* also include when a researcher outside of the country access the personal data within the country. It *could* also include putting data onto a cloud where the server is not hosted in the country. Whether it does or not is not yet settled law.

In addition to meeting the provisions set out in the law, there must be a ground under which the transfer can take place. They are:

*RA to explain how the transfer may take place. Refer back to the guide already drafted but ensure that sufficient detail is provided. Examples may be:*

1. *Whether the country to which the data is being sent has been explicitly been approved by a national authority/ministerial order as having a similar level of protection (RA to insert link to where list of countries can be found).*
2. *A country has a similar level of protection (assessment to be made by the data controller/responsible party)*
3. *The consent of the data subject to the transfer of the data to the specific country has been provided.*
4. *Transfer subject to a legally binding agreement e.g., data transfer agreement.*
5. *……*

1. For more on this point the following may be of interest: [Re‐identifiability of genomic data and the GDPR: Assessing the re‐identifiability of genomic data in light of the EU General Data Protection Regulation: EMBO reports: Vol 20, No 6 (embopress.org)](https://www.embopress.org/doi/full/10.15252/embr.201948316). [GDPR Brief: can genomic data be anonymised? – GA4GH](https://www.ga4gh.org/news_item/can-genomic-data-be-anonymised/). [Privacy in Genomics (genome.gov)](https://www.genome.gov/about-genomics/policy-issues/Privacy) [↑](#footnote-ref-1)
